# Robustness of Next Generation Sequencing on Older Formalin-Fixed Paraffin-Embedded Tissue

**DOI:** 10.1371/journal.pone.0127353

**Published:** 2015-07-29

**Authors:** Danielle Mercatante Carrick, Michele G. Mehaffey, Michael C. Sachs, Sean Altekruse, Corinne Camalier, Rodrigo Chuaqui, Wendy Cozen, Biswajit Das, Brenda Y. Hernandez, Chih-Jian Lih, Charles F. Lynch, Hala Makhlouf, Paul McGregor, Lisa M. McShane, JoyAnn Phillips Rohan, William D. Walsh, Paul M. Williams, Elizabeth M. Gillanders, Leah E. Mechanic, Sheri D. Schully

**Affiliations:** 1 Division of Cancer Control and Population Sciences (DCCPS), National Cancer Institute, 9609 Medical Center Drive, Rockville, MD 20850, United States of America; 2 Molecular Characterization and Clinical Assay Development Laboratory, Leidos Biomedical Research Inc. and Frederick National Laboratory for Cancer Research, Frederick, MD 21702, United States of America; 3 Division of Cancer Treatment and Diagnosis (DCTD), National Cancer Institute, 9609 Medical Center Drive, Rockville, MD 20850, United States of America; 4 USC Keck School of Medicine, University of Southern California, 1441 Eastlake Ave. NOR 4451A, 9175 Los Angeles, CA 90089–9175, United States of America; 5 University of Hawaii Cancer Center, University of Hawaii, 701 Ilalo Street Honolulu, HI 96813, United States of America; 6 Department of Epidemiology, College of Public Health, 145 North Riverside Dr., The University of Iowa, Iowa City, IA 52242, United States of America; UT MD Anderson Cancer Center, UNITED STATES

## Abstract

Next Generation Sequencing (NGS) technologies are used to detect somatic mutations in tumors and study germ line variation. Most NGS studies use DNA isolated from whole blood or fresh frozen tissue. However, formalin-fixed paraffin-embedded (FFPE) tissues are one of the most widely available clinical specimens. Their potential utility as a source of DNA for NGS would greatly enhance population-based cancer studies. While preliminary studies suggest FFPE tissue may be used for NGS, the feasibility of using archived FFPE specimens in population based studies and the effect of storage time on these specimens needs to be determined. We conducted a study to determine whether DNA in archived FFPE high-grade ovarian serous adenocarcinomas from Surveillance, Epidemiology and End Results (SEER) registries Residual Tissue Repositories (RTR) was present in sufficient quantity and quality for NGS assays. Fifty-nine FFPE tissues, stored from 3 to 32 years, were obtained from three SEER RTR sites. DNA was extracted, quantified, quality assessed, and subjected to whole exome sequencing (WES). Following DNA extraction, 58 of 59 specimens (98%) yielded DNA and moved on to the library generation step followed by WES. Specimens stored for longer periods of time had significantly lower coverage of the target region (6% lower per 10 years, 95% CI: 3-10%) and lower average read depth (40x lower per 10 years, 95% CI: 18-60), although sufficient quality and quantity of WES data was obtained for data mining. Overall, 90% (53/59) of specimens provided usable NGS data regardless of storage time. This feasibility study demonstrates FFPE specimens acquired from SEER registries after varying lengths of storage time and under varying storage conditions are a promising source of DNA for NGS.

## Introduction

The identification of cancer predisposition genes provided insights into mechanisms of cancer development and suggests possible targets for cancer therapy [1]. Due to reduced costs of Next-Generation Sequencing (NGS) technologies, e.g., Whole Exome Sequencing (WES), NGS is becoming an integral means to more comprehensively interrogate large numbers of genes in population-based and clinical studies [2–11].

Several large scale projects, including The Cancer Genome Atlas (TCGA)[12] and the International Cancer Genome Consortium (ICGC)[13] were developed to characterize the genomic landscapes of different cancers. These projects employed NGS technology for detection of somatic mutations predominately using fresh-frozen tissue specimens. However, these projects also successfully utilized a limited number of formalin-fixed paraffin-embedded (FFPE) tissue specimens prepared using uniform methods under strict quality control criteria. Furthermore, a recent proof-of-concept study demonstrated that NGS on FFPE tissue could be used to help guide precision cancer medicine [14]. However, for NGS to be useful in clinical settings and for population-based studies, the utility of FFPE tissue specimens collected and processed in non-uniform manners via routine clinical settings needs to be confirmed.

Several studies demonstrated, using small numbers of FFPE tissue specimens from variable sources, that NGS using FFPE tissue is feasible [11,15–20]. Many of these were conducted by micro-dissecting FFPE tissue [11,15,20] or coring areas of high tumor content to enrich tumor material [19]. These techniques are labor-intensive, and may not be realistic for large scale projects. It remains to be seen whether archival FFPE specimens, prepared in numerous pathology labs under varying laboratory conditions and stored for varying lengths of time, are suitable for NGS.

The Surveillance, Epidemiology, and End Results (SEER) cancer registries cover approximately 28% of the United States population, providing high quality demographic, clinical, pathologic, and survival data. In three of the SEER registries, annotated FFPE tumor tissue specimens are available for research use through established Residual Tissue Repositories (RTR)[21,22]. Development of population-based biospecimen research capacity in SEER offers opportunities for unbiased sampling and collection of robust samples.

The main objective of this study was to determine whether DNA obtained from FFPE tissues archived in SEER RTRs is of sufficient quantity and quality for WES and examine the effect of storage time. Resulting WES data were compared with TCGA findings to assess whether similar results can be obtained [3].

## Materials and Methods

### Subject/specimen selection

**Fig 1:**
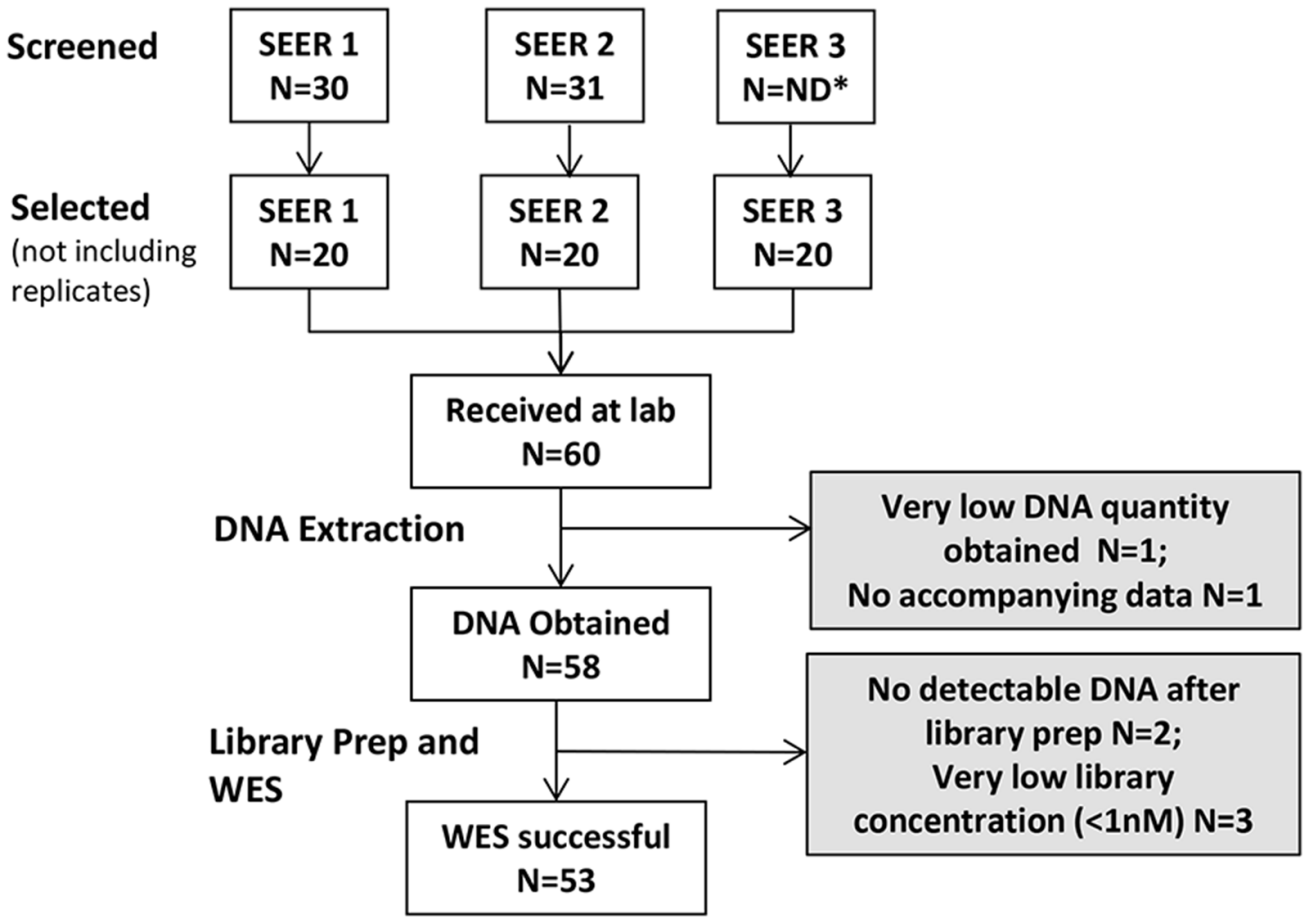
Workflow for the FFPE tissues. Three SEER RTRs selected cases that would likely meet the study criteria from their database. Cases were screened for histology/morphology, tumor nuclei content, and necrotic cell content to select cases meet the study criteria. The RTRs screened 30 or 31 cases; one RTR did not keep track of the number of cases they screened. Each RTR selected and sent 20 cases that met study criteria to the lab. One case was excluded from analyses due to incomplete information regarding storage time. 59 cases were considered for subsequent analyses. DNA was successfully extracted from 58 cases. WES was successful on 53 of the cases.

59 FFPE tissue sections (distinct cases) were sent to the Molecular Characterization and Clinical Assay Development Laboratory (MoCha) at Frederick National Laboratory for Cancer Research (Fig 1). As specimens were retrospectively collected by the RTRs from multiple medical facilities and pathology labs within each of the three catchment areas, fixation times/conditions and storage conditions were unknown. Tissues were from high-grade serous ovarian adenocarcinomas (ICD-O-3 Topography code: C56.9; Morphology codes: 8441/3, 8460/3, 8461/3) and storage time ranged from 3 to 32 years (Table 1) based on decade when tissue was resected. This cancer type was selected because it is a rare, yet aggressive cancer and has high observed frequency of mutations in *TP53* (90% of tumors, automated detection of *TP53*), which was used as a positive control [3]. This study was approved by institutional review boards at participating cancer registries (University of Southern California Health Sciences Campus, The University of Iowa and the University of Hawaii) and at the National Cancer Institute (NCI). The study was determined to be exempt from IRB review under 45 CFR 46.101(b)(4) for the use of coded or coded but unlinked tissue blocks from the SEER registries. No contact with subjects was made for this study.

**Table 1:** FFPE specimens selected for analysis.

	Specimen Storage Time				
	3–12 years	13–22 years	23–32 years	Replicates*	Total
**SEER site 1**	8	9	3	4	24
**SEER site 2**	4	11	5	3	23
**SEER site 3**	1	11	7	0	19
**Total**	13	31	15	7	66

*For seven cases two separate specimens were prepared and sent to the laboratory.

Each SEER registry conducted a pathology review of lead and trail sections flanking the 5 sections from each tissue block to determine whether tissue was consistent with the selection criteria (high-grade serous ovarian adenocarcinoma, ≥ 50% of cells with nuclei consistent with malignant cells, and ≤ 50% of cells were necrotic); approximately 30 cases from each registry were reviewed to ultimately select 20 cases that met study criteria (Fig 1). For each case identified as meeting the study criteria, five 10-micron sections were placed in a sterile tube.

### Laboratory FFPE tissue handling

Upon receipt of the specimens, the lab assessed tissue quality by conducting gross QC checks of tissue sections (e.g., check for damaged FFPE curls) and performed additional pathology review. The NCI-conducted pathology review determined whether the tissue was consistent with the tissue selection criteria. H&E slides scanned into the Aperio System were reviewed for histology and tumor content by one of two pathologists.

### DNA/RNA extraction and quantity and quality assessment

Qiagen All Prep FFPET kit was used to purify DNA and RNA from each specimen. DNA was quantified and quality checked by Nanodrop spectrophotometer (OD 260 and 280) and Qubit fluorometer. In addition, the Kapa Human Genomic DNA Quantification and QC Kit (i.e. KapaQC)[23] were used to assess Q129/41 ratio as a measure of DNA quality prior to library preparation.

### Whole exome sequencing

WES libraries were constructed using Agilent Sure Select Whole Exome Library Kit with bait v4 (capture size of 51 megabases) and were subsequently sequenced by Illumina Hiseq 2000 sequencer. 600ng (Qubit quantified) FFPE DNA per sample was fragmented into 150-200bp by Covaris E220 sonication prior to library construction. For samples with less than 600ng (S2 Table), all the extracted DNA was used. Constructed libraries were quality checked using a Agilent Bioanalyzer and quantified using Kapa library Quantification kit. Sequence quality metrics, including target exome coverage, average read depth, percent of duplication (how many reads that are mapped to the exact same position), and Transition (Ti)/Transversion (Tv) ratio, were calculated for control (Hapmap CEPH, NA12878, flash frozen (FF) DNA) and SEER specimens. Two metrics were used when comparing to TCGA ovarian cohort: coverage of 76% of the target area and depths of at least 14x for discovery (“x” = number of reads). The success of sequencing assay was defined as having a non-failed final library (>200bp) and covering at least 50% of the target at 20x.

### Use of replicates

For seven cases, two different sections, or replicates, were prepared and sent to the lab for analysis (Table 1). The ages of these samples range from 7 years to 24 years. The lab performed each study procedure on these sections in a blinded fashion. The sections were then used to assess consistency of results from DNA isolation and library preparation procedures.

### Statistical Analysis

Exact confidence intervals for the sequencing success rate were computed using the Clopper and Pearson method [24]. Tests for linear trend in success rate by age group were performed using the Cochran-Armitage test for trend. Associations of continuous quality control (QC) measures with specimen age were estimated with linear regression. Model-based standard errors for regression coefficients were used for confidence intervals and Wald tests. This approach assumed that error variances were constant as a function of specimen age, independent, and identically distributed. No noticeable departures from this assumption were encountered upon graphical examination. Analyses were performed with R version 3.0.3 software (R Foundation for Statistical Computing). No adjustments to confidence intervals or P values were made for multiple comparisons.

## Results

### Quantity and Quality of DNA from the FFPE Tissue

**Fig 2:**
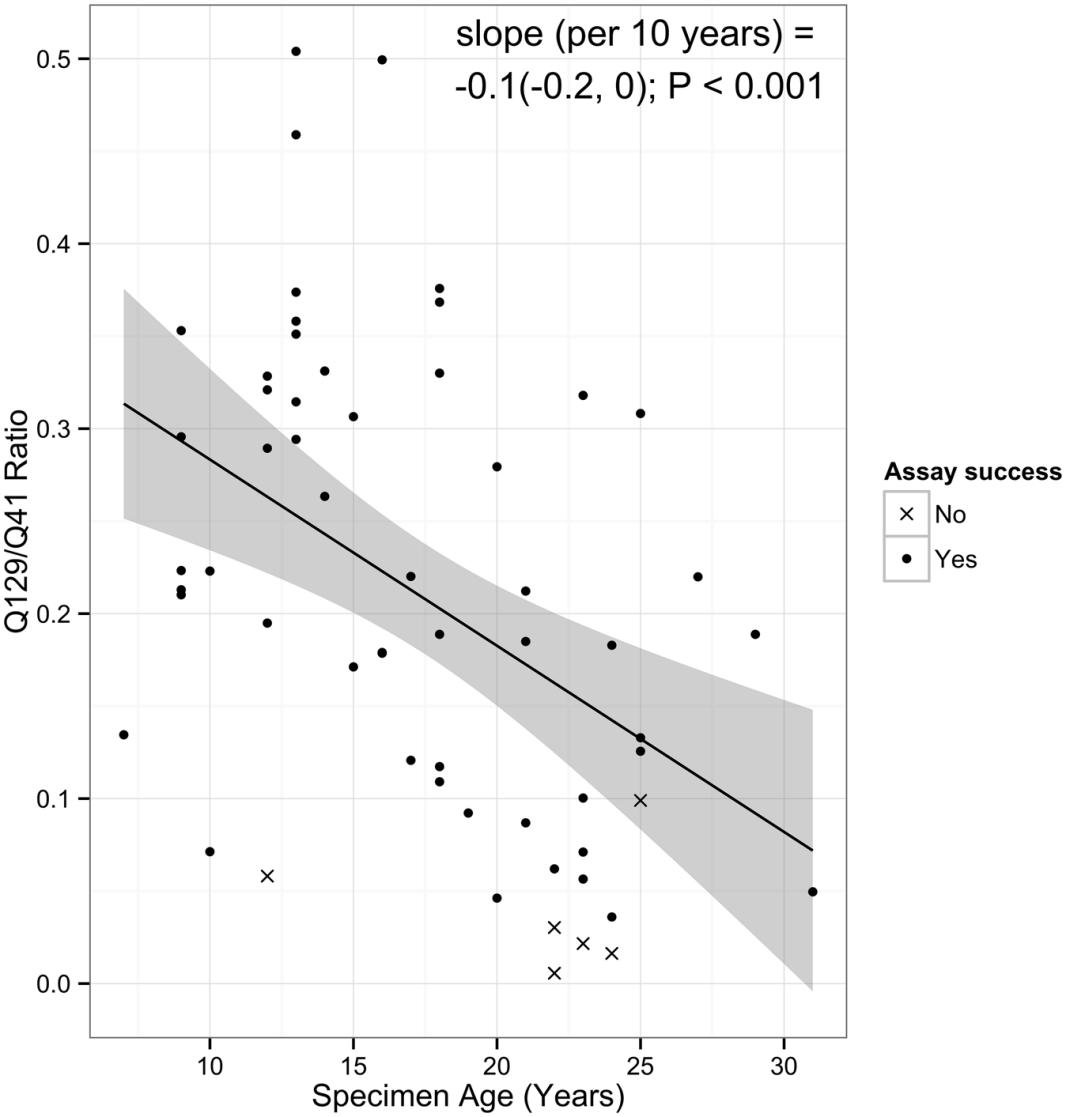
Association between specimen storage time and the Q129/41 ratio. The solid line indicates the estimated linear relationship between age and Q129/41 ratio. The shaded area denotes pointwise 95% confidence intervals of the conditional mean. Cases successful through the entire WES workflow (DNA extraction through WES sequencing) are denoted as circles (N = 53); unsuccessful cases are denoted as X’s (N = 6).

All tissue curls were received in good condition at the MoCha lab. The NCI-conducted pathology review verified the majority of tissues met desired selection criteria (Table 2). Of 59 unique specimens, one sample yielded very minimal DNA, and was considered to have failed DNA extraction; however, the Q129/41 ratio was used for age group correlation. An average of 3.7μg DNA was observed in the 58 samples that yielded DNA. Fifteen of 59 tissues (25.4%) had poor DNA quality as determined by the KapaQC result (Q129/41<0.1), while 8.47% had very poor DNA quality (Q129/41<0.04) (Table 3, Fig 2).

**Table 2:** Pathology review results of FFPE tissues for serous ovarian adenocarcinoma received at the NCI lab.

	# of Tissues		
	Yes	No	% Discordance with how the registry designated the tissue
**>50% tumor nuclei**	50	9	15%
**< = 50% necrosis**	55	4	7%
**High-grade**	56	3*	5%

*Two pathologists at NCI reviewed these 3 cases and agreed the tissue sections provided did not show sufficient evidence of being high-grade.

**Table 3:** DNA quantity and quality metrics by storage time of specimen.

	Mean (Standard Deviation) by Storage Time				Difference (95% CI)*,10 Years of Storage	P-value, Trend
	Overall(N = 59)	3-12Years(N = 13)	13-22Years(N = 31)	23–32 Years(N = 15)		
**DNA yield(μg)** ^**$**^ **(measured by Qubit)**	3.7 (3.2)	3.8 (3)	4.6 (3.4)	1.7 (2.1)	-2.1 (-3.5 to -0.7)	P = 0.003
**A260/280 ratio**	2.1 (1.2)	2.0 (0.3)	2.0 (0.5)	2.6 (2.3)	0.3 (-0.3 to 0.9)	P = 0.288
**Q129/41 ratio**	0.21(0.13)	0.22 (0.09)	0.24 (0.14)	0.13 (0.1)	-0.1 (-0.15 to -0.05)	P < 0.001

**DNA yield (μg) (measured by Qubit):** Extracted DNA concentration was measured using Qubit fluorometer using dsDNA BR Assay Kits from Life Technologies Inc. and total DNA yield for the sample were calculated.

**A260/280:** Absorbance at 260 nm and 280 nm were measured using Nanodrop for extracted DNA from each sample and expressed as a ratio.

**KapaQC Q129/41 ratio:** Kapa Human Genomic DNA Quantification and QC Kit [23] was used to measure the quality of the genomic DNA isolated from these FFPE samples. This ratio is a measure of fragmentation of the genomic DNA extracted from the sample.

*CI = confidence Interval. Overall means and standard deviations for quality measures and by storage time, differences per 10 years of storage and 95% confidence intervals estimated by linear regression on continuous time. P-values are for Wald tests of time coefficients in regression models. A slope of zero indicates no association with specimen storage time. P-values are not adjusted for multiple comparisons.

^$^ 1 sample, age group 13–22 years, is missing DNA yield because no DNA remained after the QC step.

DNA yield and quality were observed to be significantly associated with storage time (p = 0.003 for yield; p<0.001 for quality; Table 3). Specimens between 3 and 12 years old (n = 13) had an average DNA yield of 3.8 μg and 15% (2/13) had poor quality DNA while none had very poor quality DNA; S2 Table. Specimens between 13 and 22 years old (n = 31) had an average DNA yield of 4.6 μg (n = 30); 19% (6/31) had poor quality DNA, and 6% (2/31) had very poor quality DNA. One specimen in this age group did not have DNA remaining after the QC step. Specimens between 23 and 32 years old (n = 15) had an average DNA yield of 1.7 μg; 47% (7/15) had poor quality DNA, and 20% (3/15) had very poor quality DNA. There were some observable differences in DNA yields and quality by SEER registry site, but they were less statistically significant (S1 Table).

**Fig 3:**
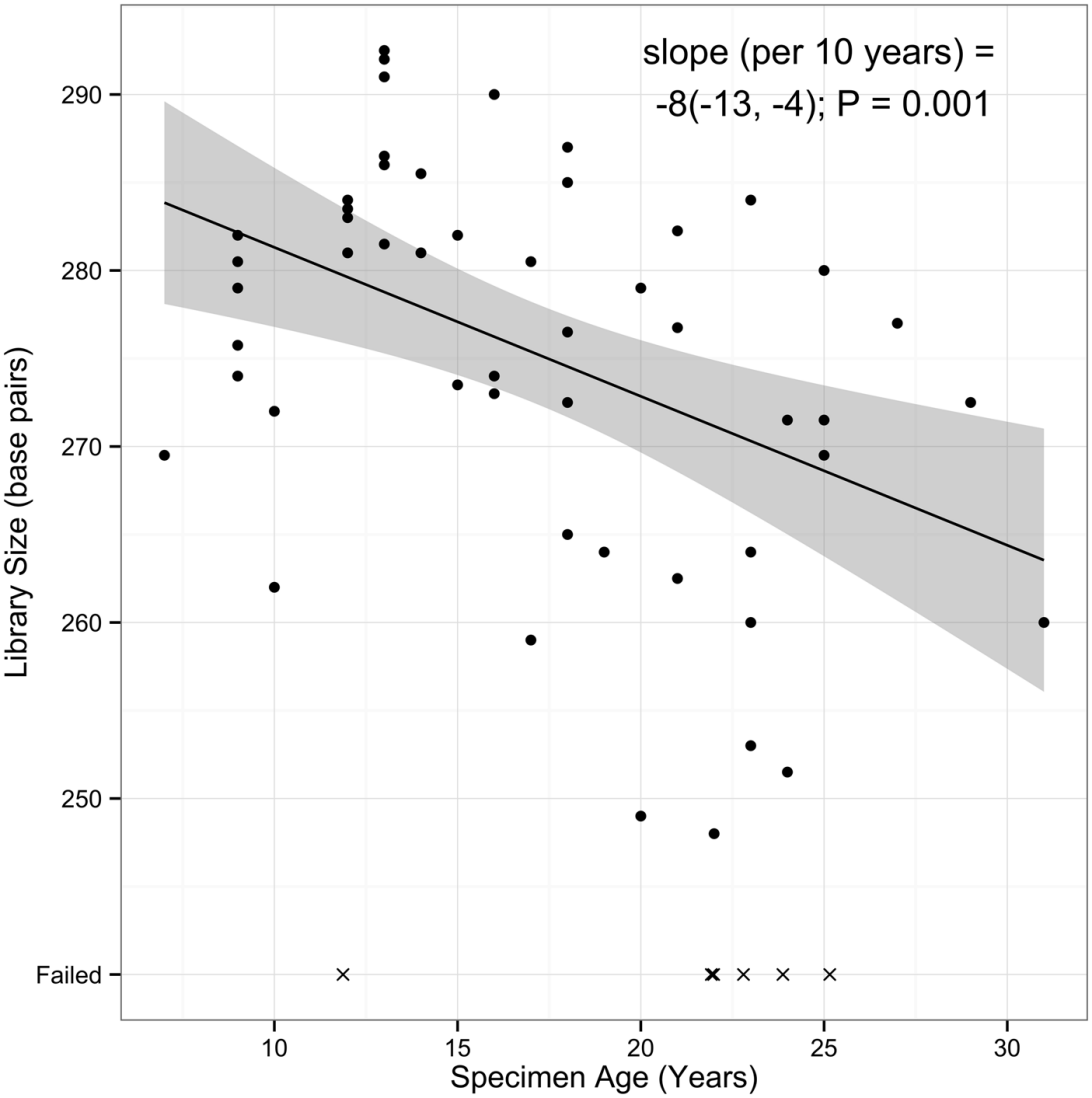
Association between specimen storage time and final library size in base pairs. Specimens that failed sequencing do not have library size values and are indicated by x. The solid line indicates the estimated linear relationship between storage time and library size (N = 59). The shaded area denotes pointwise 95% confidence intervals for the conditional means. Cases successful through the entire WES workflow (DNA extraction through WES sequencing) are denoted as circles (N = 53); unsuccessful cases are denoted as X’s (N = 6). Failed assays were not used to estimate the linear trend.

After completion of the library preparation protocol, two of the 58 specimens (3.4%) did not have detectable DNA at the final quantification step; one was in the 3 to 12 and one in the 23 to 32 storage-year categories. Three specimens had very low final library concentrations (<1nM); one was in the 13 to 22 and two in the 23 to 32 storage-year categories. However, those five specimens were sequenced to determine if there was any information to be collected to set the thresholds for specimens with the lowest acceptable quality. The average fragment size in final libraries was associated with specimen storage time (ten-year difference in specimen age was associated with an approximately 9bp lower library; Fig 3 and Table 4).

**Table 4:** Whole Exome Sequencing QC metrics by duration of specimen storage.

	Mean (Standard Deviation) by Storage Time				Difference (95% CI), 10 Years of Storage	P-value, Trend
	Overall (N = 53)	3–12 Years (N = 12)	13–22 Years (N = 29)	23–32 Years (N = 12)		
**Final Library Size (bp)**	275.3 (11.3)	277.2 (6.7)	277.6 (12.1)	267.9 (10.3)	-8.5 (-13.4 to -3.5)	P = 0.001
**% Target Covered 20x**	86.2 (7.9)	89.7 (2.8)	86.3 (8.3)	82.2 (9)	-6.1 (-9.5 to -2.6)	P < 0.001
**Average Read Depth (x)**	112.1 (48.4)	128.2 (35.5)	116 (51)	86.8 (46.8)	-39.1 (-60 to -18.2)	P < 0.001
**Percent Duplication**	33.6 (20.9)	26.6 (13.9)	34.1 (21.5)	39.5 (24.5)	9.0 (-0.9 to 18.8)	P = 0.074
**Ti/Tv Ratio**	2.48 (0.15)	2.43 (.07)	2.45 (.08)	2.61 (0.24)	0.12 (.06 to 0.18)	P < 0.001

**Final Library Size (bp):** This QC metric represents the peak size from BioAnalyzer electropherogram traces of the final exome library from each sample using the Agilent BioAnalyzer High Sensitivity DNA kit.

**% Target Covered at 20x:** The percentage of all target bases achieving 20X or greater read depth. This metric measures the efficiency of the exome capture. DNA samples with poor quality samples tend to have lower % target covered.

**Average Read Depth:** Average read depth in the target region.

**Percent Duplication:** Percent of reads originating from same fragment of the library. These duplicated reads may indicate bias originating from sample quality, library amplification etc. DNA samples with poor quality samples tend to have higher percent duplication.

**Ti/Tv Ratio:** This is ratio of transitions (single nucleotide substitutions with the same type of nucleotide, e.g., pyrimidine to pyrimidine (C<>T) or purine to purine (A<>G)) to transversions (single nucleotide substitutions with the different type of nucleotide, i.e., pyrimidine to purine or vice versa (A<>T etc.). FFPE DNA samples tend to have higher Ti/Tv ratio due to chemical crosslink and modification.

CI = confidence Interval. Overall means and standard deviations for quality measures and by storage time, differences per 10 years of storage and 95% confidence intervals estimated by linear regression on continuous time. P-values are for Wald tests of time coefficients in regression models. A slope of zero indicates no association with specimen storage time. P-values are not adjusted for multiple comparisons.

Replicates performed similarly in DNA isolation and library preparation (S2 Table). Correlation coefficients between measurements on replicates were 0.60 for library size, -0.12 for percent target coverage, 0.31 for average read depth and 0.73 for Ti/Tv ratio. Paired t-tests comparing replicates failed to yield significant differences (p-values>0.25).

### Whole exome sequencing

WES was conducted on a total of 58 unique specimens plus seven replicates. Storage time was associated with percent target coverage (p<0.001), read depth (p<0.001) and Ti/Tv ratio (p<0.001). Registry site also correlated with sequencing metrics (percent target coverage p = 0.02, read depth p = 0.03, percent duplication p<0.001; S1 Table). Five specimens failed sequencing QC metrics due to low target region coverage (< 50%) and low read depth (20x) (S2 Table). These five specimens yielded no (n = 2), or very low (n = 3), final libraries (Fig 3, S2 Table), indicating the library yield provides an in-process QC check for identifying poorly performing DNA. Additionally, the three specimens with very low final library concentrations had KapaQC Q129/41 ratios less than 0.03 (Fig 2), indicating KapaQC is also a good indicator of poorly performing DNA.

**Fig 4:**
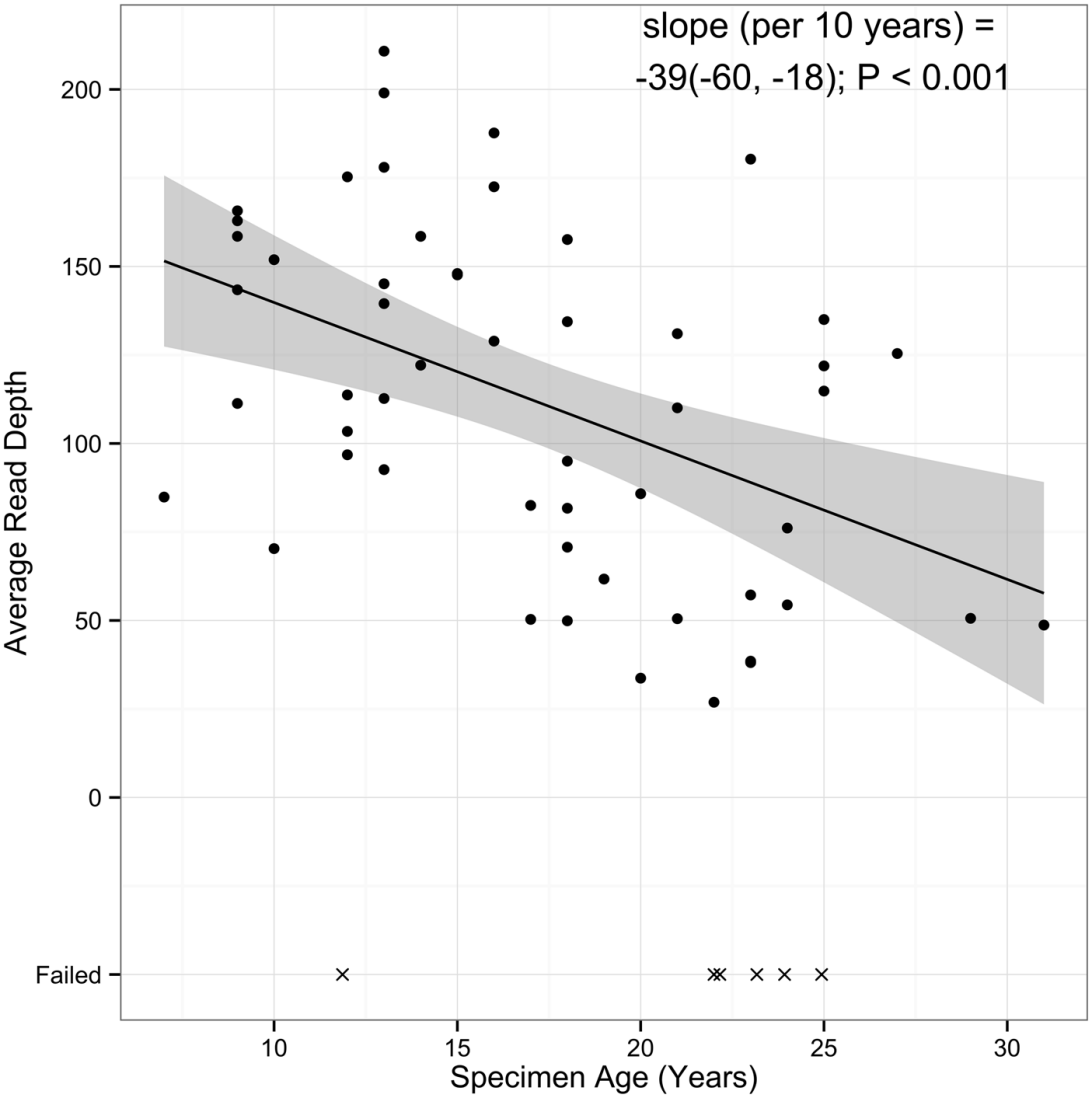
Association between specimen storage time and average read depth. The solid line indicates the estimated linear relationship between age and average read depth. The shaded area denoted pointwise 95% confidence intervals of the conditional mean. Cases successful through the entire WES workflow (DNA extraction through WES sequencing) are denoted as circles (N = 53); unsuccessful cases are denoted as X’s (N = 6). Failed assays were not used to estimate the linear trend.

For the remaining 53 unique specimens, average read depth was 112x, and was statistically significantly different among specimens by storage time (p<0.001; Table 4, Fig 4). Percent of target covered was also associated with storage time (p<0.001; Table 4). A ten-year difference in specimen age was associated with a 39x lower average read depth and 6% lower average percent target covered. Although, there is correlation of specimen age with read depth and target coverage, the results were still acceptable for data analysis. Of the 53 specimens, percent duplication increased from an average of 27% for specimens stored the shortest time to 40% for specimens stored the longest, indicating the uniquely mapped reads decreased from 73% to 60% over additional 10 years of sample storage time.

The Ti/Tv ratios were also calculated for each FFPE specimen and compared to NA12878. The Ti/Tv ratio for this normal FF specimen was 2.4, consistent with previous results. The mean Ti/Tv ratio for the 53 successful FFPE specimens was 2.5 (Table 4). Specimens with a Ti/Tv ratio on the high end of this range (i.e., >2.7) were also of poor quality and very low quality as determined by the KapaQC assay.

Overall, out of 59 independent specimens received, 53 yielded DNA of sufficient quantity and quality and were successful in the WES assay. Thus, our estimate of success probability is 89.8% (Exact 95% CI: 79% to 96%). The overall success rate was not significantly associated with time of specimen storage, p = 0.366 (Table 5) nor with SEER registry site (p = 0.693) (Table 6).

**Table 5:** Success of whole exome assay by formalin-fixed paraffin-embedded specimen storage time.

Age Range in Years	Assay Failed	Assay Successful*	Total	Proportion successful	95% Confidence Interval
**3 to 12**	1	12	13	0.92	0.64 to 0.99
**12 to 22**	2	29	31	0.94	0.79 to 0.99
**22 to 32**	3	12	15	0.80	0.52 to 0.99

* Sequencing assay success defined as having a non-failed final library size and percent target coverage of 50% with a minimum of 20x coverage.

The p-value for a linear test for trend of success probability by specimen storage time equals 0.366.

**Table 6:** Success of whole exome assays by SEER Registry sites.

Registry	Assay Failed	Assay Successful*	Total	Percent Success	95% Confidence Interval
**SEER site 1**	1	19	20	0.95	0.75 to 0.99
**SEER site 2**	2	17	19	0.89	0.67 to 0.99
**SEER site 3**	3	17	20	0.85	0.62 to 0.97

* Sequencing assay success defined as having a non-failed final library size and percent target coverage of 50% with a minimum of 20x coverage.

The p-value for a linear test for trend of success probability by site equals 0.693.

### Comparison to TCGA results

Twenty most frequently mutated genes were described in a reanalysis of TCGA ovarian serous adenocarcinoma data [25]. In the present study, we found 15 of those genes harbored at least one non-silent mutation in the SEER FFPE samples, with results comparable to the TCGA reanalysis (S3 Table). For example, we found *TP53* mutations were in 75% (40/53) of the tissues, which was comparable to what was found with TCGA (*TP53* mutations in 87% (276/316) of the patients).

## Discussion

All but one of 59 unique specimens (98%) yielded DNA of suitable quality to make a sequencing library. Overall, 90% of the specimens yielded successful WES assay results. While older specimens tended to have lower DNA sequencing quality, defined by insert library size and average read depth, the vast majority of specimens stored for 22 to 32 years (80%) yielded successful WES assay results. Although, there is a significant correlation between specimen age and library fragment size, Ti/Tv ratio, and read depth, this is unlikely to be meaningful in terms of sequencing data mining for biological insight. The observation of high reproducibility in NGS quality metrics from seven replicated samples indicated that aged, archived FFPE specimens can produce reproducible NGS results. These findings support the use of archival FFPE tissues drawn from the population to conduct hypothesis-driven research such as studies of potential therapeutic targets and biomarkers associated with prognosis.

Failures to gain usable sequence in the WES workflow can largely be predicted by the KapaQC assay (Q129/41 ratio) prior to commencement of library preparation (Fig 2). Up-front screening by KapaQC could identify specimens likely to perform poorly in sequence analysis. Such poor quality specimens could be removed from further study or if used for study the variants identified in these poor quality specimens.

The FFPE fixation process is known to introduce molecular artifacts compared with frozen tissue. As in the present study, Hedegaard and colleagues [22] reported degradation in DNA library size and target coverage associated with increasing storage time. This could be influenced by temporal changes in fixation practices [22].

We have observed a slightly higher Ti/Tv ratio in the FFPE clinical specimens (avg. 2.5) compared to our FF control DNA (2.4). These results are difficult to fully interpret, but it is consistent with previous reports [20,26] that suggest DNA from FFPE specimens have higher levels of Ti/Tv ratios compared to freshly prepared DNA possibly due to deamination of cytosine residues [27]. It is interesting to note that the average Ti/Tv ratio increased with the age of the FFPE block (Table 4).

One of the goals of this study was to determine whether specific FFPE tissue could be obtained from SEER. We demonstrated high concordance between the NCI and SEER registry pathology reviews of the tissues. Yet, there was 15% discordance with regards to tumor nuclei, 7% for necrosis, and 5% for high-grade assessment. This indicates the importance of multiple pathology reviews to more precisely define the tissue section being analyzed and a need for standardization, if possible. It also suggests that caution must be used when analyzing sections of tissue to ensure that they contain the intended disease tissue; three cases were determined by NCI pathologists to not be high-grade serous ovarian adenocarcinomas, likely due to the section of tissue not harboring that particular histology.

This study had strengths and limitations. A strength was the ability to acquire and analyze FFPE tissue meeting specific inclusion criteria and had been stored for variable time periods. However, to do so required screening approximately 33% additional cases than were needed. A limitation was the lack of FF tumor tissue from the same patients to compare FFPE results. However, several studies have provided evidence showing FFPE is an acceptable alternative when FF tissue is not available for NGS [19,22,28]. When we compared the FFPE data with TCGA data, *TP53* mutations were detected in a similar proportion of FF tissue (87% in TCGA) and SEER FFPE tissue (75% in the present study). While TCGA collected both high and low grade serous ovarian adenocarcinoma, the present study only used high grade serous tumors. Given the differences in pathology, tissue type, and storage time between these two studies, similar observation in highly mutated genes in such a large number of FFPE tissue (S3 Table), suggests that FFPE is an acceptable alternative.

## Conclusion

In summary, this study demonstrates that FFPE specimens acquired from SEER catchments after varying lengths of time and under varying storage conditions have potential value as sources of DNA for NGS. Expanding access for investigators to registry-based FFPE materials could have merit as a means of advancing hypothesis-driven cancer research. While FFPE specimens stored for many years may have poorer quality DNA and yield smaller library inserts, lower average read depths, and higher duplication than more contemporary specimens, usable WES data were obtained for the vast majority of FFPE specimens, regardless of storage time. Researchers conducting population-based studies will find this data encouraging and can draw upon our findings to facilitate design of studies with NGS analyses of somatic mutations using FFPE tissue from existing collections handled under typical conditions in health care settings.

## Supporting Information

S1 Table Summary of DNA and sequencing QC metrics by SEER registry site providing the specimens. (DOC)

S2 Table DNA preparation and sequencing metrics for the unique specimens as well as replicates. (XLS)

S3 Table Comparison of most significantly mutated genes in TCGA and present study. (XLSX)
