# Supplementary material for: Robustness of Next Generation Sequencing on Older Formalin-Fixed Paraffin-Embedded Tissue
Source: PLoS One. 2015 Jul 29;10(7):e0127353. doi: 10.1371/journal.pone.0127353 (PMC4519244; doi:10.1371/journal.pone.0127353)
Supplement: S1 Table — (DOC) [file pone.0127353.s001.doc]

**Supplemental Table 1. Summary of DNA and sequencing QC metrics by SEER registry site providing the specimens***

|  | **Overall Mean (Standard Deviation) and by Registry** | | | | **P-value,**  **Registry Differences** |
| --- | --- | --- | --- | --- | --- |
|  | **Overall** | **SEER site 1** | **SEER site 2** | **SEER site 3** |  |
| ***DNA QC Metrics*** | **N=59** | **N=20** | **N=19** | **N=20** |  |
| **DNA yield (**μ**g) (measured by Qubit)** | 3.7 (3.2) | 4.4 (2.9) | 2.3 (2.7) | 4.3 (3.6) | P = 0.072 |
| **A260/280** | 2.15 (1.25) | 2.04 (0.25) | 2.62 (0.21) | 1.81 (0.3) | P = 0.114 |
| **KapaQC Q129/41 ratio** | 0.21 (0.12) | 0.24 (0.12) | 0.17 (0.13) | 0.21(0.12) | P = 0.185 |
| ***Library QC Metrics*** | **N=53** | **N=19** | **N=17** | **N=17** |  |
| **Final Library Size (bp)** | 275.3 (11.3) | 278 (10.1) | 270.1 (13.4) | 277.5 (8.9) | P = 0.067 |
| ***Sequencing QC Metrics*** | **N=53** | **N=19** | **N=17** | **N=17** |  |
| **% Target Covered at 20x** | 86.2 (7.9) | 88 (4.3) | 81.9 (10.9) | 88.5 (5.7) | P = 0.02 |
| **Average Read Depth** | 112.1 (48.4) | 113.6 (49) | 89.7 (47.6) | 133 (40.6) | P = 0.029 |
| **Percent Duplication** | 33.6 (20.9) | 26.3 (12.7) | 49 (25.5) | 26.4 (14.8) | P < 0.001 |
| **Ti/Tv Ratio** | 2.48 (0.15) | 2.46 (0.14) | 2.52 (0.12) | 2.47 (0.17) | P = 0.443 |

* P-values for differences by site are from the likelihood ratio test. ^$^ 1 sample, SEER site 2, is missing DNA yield because no DNA remained after the QC step.
